# Peroxisome Proliferator-Activated Receptors in HBV-Related Infection

**DOI:** 10.1155/2009/145124

**Published:** 2009-04-09

**Authors:** Laurent Dubuquoy, Alexandre Louvet, Antoine Hollebecque, Philippe Mathurin, Sébastien Dharancy

**Affiliations:** ^1^Institute National de la Santé et de la recherche Médicale INSERM, U795, 59037 Lille, France; ^2^University Lille 2, 59045 Lille, France; ^3^Service des Maladies de l'Appareil Digestif et de la Nutrition, Hôpital Huriez, CHRU Lille, 59037 Lille, France

## Abstract

Thirty years after its discovery, the hepatitis B virus (HBV) still remains a major global public health problem. Worldwide, two billion subjects have been infected, 350 million have a chronic infection and more than 600 000 die annually of HBV-related liver disease or hepatocellular carcinoma; new infections occur because of the presence of a large reservoir of chronic carriers of the virus. Since a decade several studies describe the interrelations between HBV and nuclear receptors and more particularly the peroxisome proliferator-activated receptors (PPARs). After a brief introduction, this review will make a rapid description of HBV incidence and biology. Then a report of the literature on the role of PPARs on viral transcription and replication will be developed. Finally, the role of HBV on PPAR*γ* expression and activity will be discussed. Concluding remarks and perspectives will close this review.

## 1. Introduction

Hepatitis B virus (HBV) infection is a major public health problem with approximately 350 million people chronically infected but the prevalence of HBV infection and patterns of transmission vary greatly throughout the world. Fifteen percent to 40% of HBV-infected patients will develop cirrhosis, liver failure, and hepatocellular carcinoma (HCC) [1]. *Hepatitis B virus was considered to be not directly cytopathic*, and the development of HCC in individuals with chronic HBV infection is a multistage, multifactorial process including the interaction between host and environmental factors. *However, a recent study suggested that elevated serum HBV DNA level (≥10 000 copies/mL) was a risk predictor of HCC independent of hepatitis B e antigen (HBeAg), serum alanine aminotransferase level, and liver cirrhosis suggesting that HVB proteins themselves may have direct effect on cellular functions* [2].

Recent data suggested the implication of nuclear hormone receptor and especially of the retinoid X receptors (RXRs) and peroxisome proliferator-activated receptors (PPARs) in the transcription and the replication of the HBV. The peroxisome proliferator-activated receptors (PPARs) *α*, *β*/*δ*, and *γ* are members of the nuclear receptor superfamily activated by fatty acids and involved in the transduction of metabolic and nutritional signals into transcriptional responses [3, 4]. Among these transcription factors, PPAR*α*/*γ* together with their obligate partner the RXR are three main nuclear receptors expressed in the liver [5–7]. However, despite strong expression in the liver, proof of an eventual role of PPARs in hepatic disease remains limited to the link between hepatic tumorigenesis and chronic administration of PPAR*α* activators in rodents [8], the development of extensive hepatic steatosis in response to fasting and delayed liver regeneration in PPAR*α* knock-out mice [9, 10], impaired expression of PPAR*α* in a murine model of alcoholic liver diseases [11], and impaired liver expression of PPAR*α* influenced by the HCV core protein during chronic hepatitis C virus infection [12].

This review will first describe the importance of HBV infection worldwide and the biology of the virus. Then the interactions between PPARs and HBV will be developed to provide a precise picture of the potential role of PPARs in HBV pathophysiology.

## 2. Hepatitis B Virus: Incidence and Prevalence

*Approximately 2 billion people have been exposed to the HBV and 350 million people are chronically infected with the virus. Each year over 1 million people die from HBV-related liver disease. The chapter below will expose the incidence and prevalence of this huge public health problem worldwide.*

The prevalence of HBV infection varies depending on the geographical area. In the Far East, the Middle East, Africa, and parts of South America, the prevalence is high, with hepatitis B surface antigens (HBsAgs) rates ranging from 8% to 15% [13]. In regions of high HBsAg endemicity, serologic evidence of prior HBV infection (anti-HBc and/or anti-HBs Ag) is almost universal in subjects without active infection. As a general rule, in these areas with high HBV endemicity the source of infection is mainly through perinatal transmission from the chronically infected mother or through infection during early childhood.

Areas of intermediate prevalence (2–7%) include Japan, parts of South America, Eastern and Southern Europe, and parts of central Asia. Areas with low HBV endemicity (prevalence of chronic infection <2%) include Northwestern Europe, North America, and Australia [14]. The source of infection in these areas is mainly through sexual contacts and needle sharing among injecting drug users, with a peak incidence in the 15–25-year-old age group.

Globally the incidence of acute HBV infection has been falling in the last decade, due to changes in behavior (e.g., increase in safe sexual practices related to HIV education efforts) and, to a lesser extent, to the introduction of effective vaccination programs [15]. Transmission of HBV via transfusion of blood and plasma-derived products has been eliminated in most countries through donor screening for HBsAg and viral inactivation procedures.

## 3. Viral Structure, Genomic Organization and Replication

*HBV is a member of the family of the hepadnaviridae, hepatotropic DNA viruses. Characteristics of these viruses are as follows: a partially double-stranded DNA, with an outer lipoprotein envelope and an inner nucleocapsid or core bearing the viral genome; a polymerase with reverse transcription activity; the massive overproduction of viral envelope proteins (e.g., HBsAg), and a relative but not absolute hepatotropism. The following chapter will briefly describe the viral structure, genomic organization and replication mode of the HBV.*

**Figure 1:**
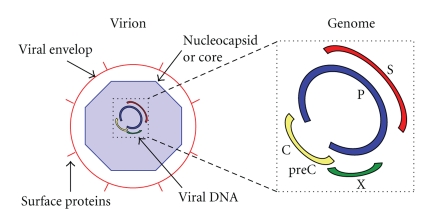
*HBV virion and genomic organization*. The HBV virion is composed of a viral envelop that contains the surface proteins, which are of different lengths (L, M, and S). The nucleocapsid or core wraps the viral DNA. The viral genome contains four open reading frames, the S that encodes for the surface protein (red), P that encodes for the viral polymerase (blue), preC and C that encode for the core (yellow) and X that encodes for the X protein (green).

HBV virions are 42 nm double-shelled particles. The genome contains four open reading frames (ORFs) (S, P, C, and X) that encode four major proteins (surface, polymerase, core, and X protein, resp.) (Figure 1). The major abundant protein on the virus surface is the HBsAg or S protein, 24 kDa in size. In the viral envelope there are two other proteins, the L—involved in binding the virus to a receptor on the hepatocyte surface—and the M protein, whose function is unknown.

The 27 nm nucleocapsid is an icosahedral symmetric structure containing 180 or 240 copies of the viral core (C) protein [16, 17], known as hepatitis B core antigen (HBcAg). The nucleocapsid contains the viral genome (Figure 1), a relaxed circular molecule that consists of a 3.2 kB minus strand and a smaller, complementary DNA (plus strand) of variable length. Circularity of HBV is maintained by hydrogen bonds between 250 bp at the two 50 ends of the plus and minus strands. The 50 ends of the DNA strands are each linked covalently to additional structures, essential for the initiation of DNA synthesis, that is, the polymerase and an oligo RNA. The viral polymerase is encoded by the P gene of the virus and is implicated in the synthesis of both strands of viral DNA through a reverse transcriptase (protein P) enzyme (RT). This RT shares sequence similarities with retroviral RT; the latter has been used in the development of antiviral drugs against HBV.

In addition to complete virions, HBV-infected hepatocytes produce in great excess two distinct subviral lipoprotein particles: the spheres, containing primarily the S protein, and the filaments, less numerous, rich in L protein. As these subviral particles contain only envelope glycoproteins and host-derived lipids, but not viral DNA; they are not infectious; nevertheless, they strongly stimulate the production of neutralizing anti-HBs antibodies. The overproduction of these particles makes it easy to diagnose HBV infection by the detection of the surface antigen in the blood.

Little is known about the earliest steps in the HBV life cycle. Virion binding to hepatocytes is mediated by a 180 kDa host protein identified as a member of the carboxypeptidase family [18]; antibodies against this protein block viral infection [19]. After direct membrane fusion uncoating of the virus allows the presentation of the nucleocapside to the cytosol. The naked viral core migrates to the nucleus where the viral genome is repaired to a covalently closed circular form (cccDNA). This cccDNA is transcribed by host RNA polymerase II to generate genomic and subgenomics stable RNAs. All viral RNAs are transported to the cytoplasm for translation yielding the viral envelope, core and preC, viral DNA polymerase, and X proteins. Finally, nucleocapsids are assembled in the cytosol; assembly requires the binding of viral polymerase (P) to a selective structure located at the 5′end of the genomic RNA. Once the P-RNA complex is formed, RNA packaging and reverse transcription begin. The replication of HBV requires an RNA intermediate followed by the synthesis of viral DNA by RT [20]. After replication is completed, viral cores are transported back into the nucleus, where they are either converted to cccDNA to maintain a stable intranuclear pool of transcriptional templates or more frequently, bud into the endoplasmic reticulum or Golgi apparatus; in this site nucleocapsidic particles are wrapped in the envelope proteins (surface, L, and M) and finally exported from the cell as full virions by vesicular transport [21].

## 4. Impact of PPAR on Viral Transcription and Replication

*Studies in hepatoma cell line HepG2 and studies on a transgenic mouse model for HBV have provided evidence for a role of PPARS in controlling viral transcription and replication.*

**Figure 2:**
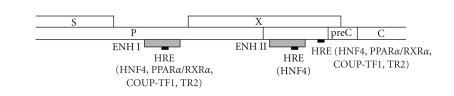
*NR regulatory region in HBV genome*. Schematic diagram of the HBV genome. The viral polymerase (P), surface proteins (S), precore (preC), core (C), and X protein (X) open reading frames are indicated by open rectangular boxes. Enhancers (ENHs) I and II are indicated by grey rectangular boxes. The hormone response elements (HREs) are indicated by small black rectangular boxes. Nuclear receptors that can bind these HREs are indicated into brackets.

HBV has a partially double-stranded DNA genome and replicates through an RNA intermediate. After infecting host liver cells, there are four HBV transcripts from four different viral promoters: Core, SPI, SPII, and X promoter. The first studies that have linked PPAR and HBV have shown the presence of hormone response elements (HREs) in the promoters of HBV genome (Figure 2). In the dedifferentiated hepatoma cell line, HepG2, it was found that the nucleocapsid and large surface antigen promoters were transactivated in the presence of hepatocyte nuclear factor 4 (HNF4) whereas the enhancer I/X gene, nucleocapsid, and large surface antigen promoters were transactivated in the presence of RXR and PPAR [22]. Characterization of the nucleocapsid promoter region demonstrated that HNF4 is the primary transcription factor binding to the regulatory region spanning nucleotides −127 to −102 whereas HNF4, RXR-PPAR heterodimers, and chicken ovalbumin upstream promoter transcription factor 1 (COUP-TF1) bind the regulatory region spanning nucleotides −34 to −7 [22]. Modulation of the level of transcription from the nucleocapsid promoter by RXR-PPAR appears to be regulated by the regulatory sequence element spanning nucleotides −34 to −7 and the HBV enhancer I region (Figure 2). Another study demonstrated that HNF4 and testicular receptor 2 (TR2) repressed synthesis of the pre-C RNA, whereas PPAR-RXR activated synthesis of the pregenomic RNA and COUP-TF1 repressed synthesis of both the pre-C and pregenomic RNAs [23].

The regulation of HBV transcription and regulation were then explored in vivo. Using an HBV transgenic mouse model, Guidotti et al. demonstrated that activation of PPAR*α* increased transcription and replication of HBV and suggested that even a modest alteration in transcription could have big impact on virus replication [24]. To point out the importance of nuclear receptors and specially PPAR*α* on the HBV replication, Tang and McLachlan have shown that ectopic expression of HNF4 and PPAR*α* was necessary and sufficient to allow HBV replication in nonhepatic cells, which is normally impossible due to the virus tropism [25].

Two studies performed in the team of McLachlan in La Jolla specified the sequences of interaction between the HBV and PPAR*α* [26, 27]. Indeed, this team has developed a transgenic mouse for a natural hepatitis B virus (HBV) variant associated with seroconversion from HBeAg to anti-HBe antibody that contains two nucleotide substitutions (A1764T and G1766A) in the proximal nuclear hormone receptor binding site in the nucleocapsid promoter. This model suggested that peroxisome proliferators may enhance viral transcription directly in a PPAR*α*-dependent manner through the nuclear hormone receptor recognition site in the enhancer I region of the HBV genome. Moreover, those mice transcribe very little precore RNA and secrete extremely low levels of HBe antigen compared with the wild-type HBV transgenic mice [26]. Analysis of HBV transcription and replication in nonhepatoma cells indicates that PPAR*α*/RXR*α* heterodimers support higher levels of pregenomic RNA transcription from the wild-type than from the variant nucleocapsid promoter, producing higher levels of wild-type than of variant replication intermediates [27]. These observations indicate that the replication of wild-type and variant viruses can be differentially regulated by the liver-specific transcription factors that bind to the proximal nuclear hormone receptor binding site of the nucleocapsid promoter.

More recent data concern approaches to counteract this nuclear receptor-induced HBV transcription and replication. Oropeza et al. showed that the nuclear receptor short heterodimer partner (SHP) inhibits the nuclear receptor-mediated HBV replication [28]. HBV replication that is dependent on HNF4 seemed considerably more sensitive to SHP-mediated inhibition than PPAR*α*/RXR*α*-directed viral biosynthesis. A nonnucleosidic compound, Helioxanthin (HE-145), was found to suppress HBV gene expression and replication in HCC cells. It was found that HE-145 selectively suppresses surface antigen promoter II (SPII) and core promoter (CP) but has no effect on surface antigen promoter I (SPI) or promoter for X gene (XP). Tseng et al. showed that HE-145 acted by decreasing the DNA-binding activity of PPAR to specific cis element of HBV promoter for core antigen [29]. Taken together, all these data provide an interesting rationale for modulating the PPAR*α*/RXR*α* heterodimer to control the HBV infection.

## 5. HBV Modulates PPAR*γ* Expression: Role in Steatosis

*Until now, two studies described a role of HBx protein on the regulation of PPAR*γ* expression and activation and one of which suggests a role in steatosis.*

In the below paragraph, we have described the role of PPAR on HBV transcription and replication. Conversely, the HBx protein of HBV modulated PPAR*γ* by protein-protein interaction. Indeed, ligand activation of PPAR*γ* has been reported to induce growth inhibition and apoptosis in various cancers including HCC. Choi and coll demonstrated that HBx counteracted growth inhibition caused by PPAR*γ* ligand in HBx-associated HCC cells [30]. They found that HBx bound to DNA binding domain of PPAR*γ* and this interaction blocked nuclear localization and binding to PPRE. HBx significantly suppressed the PPAR*γ* mediated transactivation.

**Figure 3:**
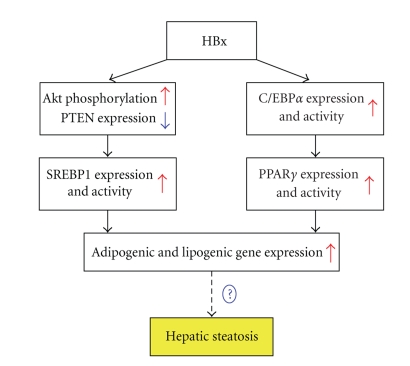
*HBx protein* could influence *liver steatosis through SREBP1 and PPAR*γ*.* Protein X of the HBV (HBx) increases the kinase AKT phosphorylation and inhibits PTEN expression that leads to increased expression and activation of SREBP1 in the liver. In another way HBx enhances C/EBP*α* that in turn induces PPAR*γ* expression and activation. Both pathways lead to an increased expression of adipogenic and lipogenic genes, which finally *could* contribute to liver steatosis.

More recent report described a positive effect of HBx protein on PPAR*γ* expression and transcriptional activity [31]. *Some* observations suggest that chronic HBV infection is associated with hepatic steatosis, which is a common histological feature of chronic infection with hepatitis C virus [32]. *Even if other report described lower frequency of steatosis in hepatitis B* [33, 34], evidence indicates that hepatic steatosis is a more vulnerable factor that leads to liver inflammation, fibrosis, and cancer. Based on these observations, Kim et al. demonstrated that overexpression of HBx induced hepatic lipid accumulation [31]. This phenomenon was accompanied by increased expression of sterol regulatory element binding protein 1 (SREBP1) and PPAR*γ*. The authors proposed that HBx could participate to hepatic steatosis during HBV infection by regulating SREBP1 and PPAR*γ* expression and activation (Figure 3) *but a direct proof remains to be obtained*.

## 6. Conclusion

HBV infection is a global health problem and recent data indicate that the HBV DNA level is a strong risk predictor of liver cirrhosis and HCC. Studies indicate the presence of hormone response elements in the promoters of HBV genome. Peroxisome proliferators may enhance HBV viral transcription directly in a PPAR*α*-dependent manner. Conversely, HBx protein of HBV is able to induce the gene expression and transcriptional activity of SREBP1 and PPAR*γ*, thereby causing hepatic lipid accumulation by increasing adipogenic and lipogenic gene expression. This regulation loop between PPAR and HBV may contribute to the progression of HBV-induced pathogenesis and the development of PPAR antagonist could represent a new therapeutic strategy.

## Abbreviations

cccDNA: Covalently closed circular DNA
COUP-TF1: Chicken ovalbumin upstream promoter transcription factor 1
HBV: Hepatitis B virus
HBcAg: Hepatitis B core antigen
HBeAg: Hepatitis B e antigen
HBsAg: Hepatitis B surface antigen
HCC: Hepatocellular carcinoma
HIV: Human immunodeficiency virus
HNF4: Hepatocyte nuclear factor 4
ORF: Open reading frame
PPAR: Peroxisome proliferator-activated receptor
RT: Reverse transcriptase enzyme
RXR: Retinoid X receptor
SHP: Short heterodimer partner
SREBP1: Sterol regulatory element binding protein 1
TR2: Testicular receptor 2

## References

[1] Lok AS (2002). Chronic hepatitis B. *The New England Journal of Medicine*.

[2] Chen C-J, Yang H-I, Su J (2006). Risk of hepatocellular carcinoma across a biological gradient of serum hepatitis B virus DNA Level. *The Journal of the American Medical Association*.

[3] Dreyer C, Krey G, Keller H, Givel F, Helftenbein G, Wahli W (1992). Control of the peroxisomal *β*-oxidation pathway by a novel family of nuclear hormone receptors. *Cell*.

[4] Mangelsdorf DJ, Thummel C, Beato M (1995). The nuclear receptor superfamily: the second decade. *Cell*.

[5] Auboeuf D, Rieusset J, Fajas L (1997). Tissue distribution and quantification of the expression of mRNAs of peroxisome proliferator-activated receptors and liver X receptor-*α* in humans: no alteration in adipose tissue of obese and NIDDM patients. *Diabetes*.

[6] Palmer CNA, Hsu M-H, Griffin KJ, Raucy JL, Johnson EF (1998). Peroxisome proliferator-activated receptor-*α* expression in human liver. *Molecular Pharmacology*.

[7] Ulven SM, Natarajan V, Holven KB, Løvdal T, Berg T, Blomhoff R (1998). Expression of retinoic acid receptor and retinoid X receptor subtypes in rat liver cells: implications for retinoid signalling in parenchymal, endothelial, Kupffer and stellate cells. *European Journal of Cell Biology*.

[8] Reddy JK, Azarnoff DL, Hignite CE (1980). Hypolipidaemic hepatic peroxisome proliferators form a novel class of chemical carcinogens. *Nature*.

[9] Hashimoto T, Cook WS, Qi C, Yeldandi AV, Reddy JK, Rao MS (2000). Defect in peroxisome proliferator-activated receptor *α*-inducible fatty acid oxidation determines the severity of hepatic steatosis in response to fasting. *The Journal of Biological Chemistry*.

[10] Anderson SP, Yoon L, Richard EB, Dunn CS, Cattley RC, Corton JC (2002). Delayed liver regeneration in peroxisome proliferator-activated receptor-*α*-null mice. *Hepatology*.

[11] Wan Y-JY, Morimoto M, Thurman RG, Bojes HK, French SW (1995). Expression of the peroxisome proliferator-activated receptor gene is decreased in experimental alcoholic liver disease. *Life Sciences*.

[12] Dharancy S, Malapel M, Perlemuter G (2005). Impaired expression of the peroxisome proliferator-activated receptor alpha during hepatitis C virus infection. *Gastroenterology*.

[13] André F (2000). Hepatitis B epidemiology in Asia, the Middle East and Africa. *Vaccine*.

[14] Lavanchy D (2005). Worldwide epidemiology of HBV infection, disease burden, and vaccine prevention. *Journal of Clinical Virology*.

[15] Centers for Disease Control and Prevention (CDC) (2007). Progress in hepatitis B prevention through universal infant vaccination—China, 1997–2006. *Morbidity and Mortality Weekly Report*.

[16] Crowther RA, Kiselev NA, Böttcher B (1994). Three-dimensional structure of hepatitis B virus core particles determined by electron cryomicroscopy. *Cell*.

[17] Böttcher B, Wynne SA, Crowther RA (1997). Determination of the fold of the core protein of hepatitis B virus by electron cryomicroscopy. *Nature*.

[18] Kuroki K, Eng F, Ishikawa T, Turck C, Harada F, Ganem D (1995). gp180, a host cell glycoprotein that binds duck hepatitis B virus particles, is encoded by a member of the carboxypeptidase gene family. *The Journal of Biological Chemistry*.

[19] Urban S, Schwarz C, Marx UC, Zentgraf H, Schaller H, Multhaup G (2000). Receptor recognition by a hepatitis B virus reveals a novel mode of high affinity virus-receptor interaction. *The EMBO Journal*.

[20] Summers J, Mason WS (1982). Replication of the genome of a hepatitis B-like virus by reverse transcription of an RNA intermediate. *Cell*.

[21] Locarnini S (2004). Molecular virology of hepatitis B virus. *Seminars in Liver Disease*.

[22] Raney AK, Johnson JL, Palmer CNA, Mclachlan A (1997). Members of the nuclear receptor superfamily regulate transcription from the hepatitis B virus nucleocapsid promoter. *The Journal of Virology*.

[23] Yu X, Mertz JE (1997). Differential regulation of the pre-C and pregenomic promoters of human hepatitis B virus by members of the nuclear receptor superfamily. *The Journal of Virology*.

[24] Guidotti LG, Eggers CM, Raney AK (1999). In vivo regulation of hepatitis B virus replication by peroxisome proliferators. *The Journal of Virology*.

[25] Tang H, McLachlan A (2001). Transcriptional regulation of hepatitis B virus by nuclear hormone receptors is a critical determinant of viral tropism. *Proceedings of the National Academy of Sciences of the United States of America*.

[26] Raney AK, Kline EF, Tang H, McLachlan A (2001). Transcription and replication of a natural hepatitis B virus nucleocapsid promoter variant is regulated in vivo by peroxisome proliferators. *Virology*.

[27] Tang H, Raney AK, McLachlan A (2001). Replication of the wild type and a natural hepatitis B virus nucleocapsid promoter variant is differentially regulated by nuclear hormone receptors in cell culture. *The Journal of Virology*.

[28] Oropeza CE, Li L, McLachlan A (2008). Differential inhibition of nuclear hormone receptor-dependent hepatitis B virus replication by the small heterodimer partner. *The Journal of Virology*.

[29] Tseng YP, Kuo YH, Hu C-P (2008). The role of helioxanthin in inhibiting human hepatitis B viral replication and gene expression by interfering with the host transcriptional machinery of viral promoters. *Antiviral Research*.

[30] Choi Y-H, Kim H-I, Seong JK (2004). Hepatitis B virus X protein modulates peroxisome proliferator-activated receptor *γ* through protein-protein interaction. *FEBS Letters*.

[31] Kim KH, Shin H-J, Kim K (2007). Hepatitis B virus X protein induces hepatic steatosis via transcriptional activation of SREBP1 and PPAR*γ*. *Gastroenterology*.

[32] Gordon A, McLean CA, Pedersen JS, Bailey MJ, Roberts SK (2005). Hepatic steatosis in chronic hepatitis B and C: predictors, distribution and effect on fibrosis. *Journal of Hepatology*.

[33] Moucari R, Asselah T, Cazals-Hatem D (2008). Insulin resistance in chronic hepatitis C: association with genotypes 1 and 4, serum HCV RNA level, and liver fibrosis. *Gastroenterology*.

[34] Wong GL-H, Wong VW-S, Choi PC-L (2009). Metabolic syndrome increases the risk of liver cirrhosis in chronic hepatitis B. *Gut*.

